# Livestock Susceptibility to Infection with Middle East Respiratory Syndrome Coronavirus 

**DOI:** 10.3201/eid2302.161239

**Published:** 2017-02

**Authors:** Júlia Vergara-Alert, Judith M.A. van den Brand, W. Widagdo, Marta Muñoz, Stalin Raj, Debby Schipper, David Solanes, Ivan Cordón, Albert Bensaid, Bart L. Haagmans, Joaquim Segalés

**Affiliations:** Centre de Recerca en Sanitat Animal, Barcelona, Spain (J. Vergara-Alert, M. Muñoz, D. Solanes, I. Cordón, A. Bensaid, J. Segalés);; Erasmus Medical Center, Rotterdam, the Netherlands (J.M.A. van den Brand, W. Widagdo, V.S. Raj, D. Schipper, B.L. Haagmans);; Universitat Autònoma de Barcelona, Barcelona (J. Segalés)

**Keywords:** Middle East respiratory syndrome, coronavirus, MERS, MERS-CoV, livestock, pig, llama, horse, sheep, animal model, reservoir, dipeptidyl peptidase-4, DPP4, viruses

## Abstract

Middle East respiratory syndrome (MERS) cases continue to be reported, predominantly in Saudi Arabia and occasionally other countries. Although dromedaries are the main reservoir, other animal species might be susceptible to MERS coronavirus (MERS-CoV) infection and potentially serve as reservoirs. To determine whether other animals are potential reservoirs, we inoculated MERS-CoV into llamas, pigs, sheep, and horses and collected nasal and rectal swab samples at various times. The presence of MERS-CoV in the nose of pigs and llamas was confirmed by PCR, titration of infectious virus, immunohistochemistry, and in situ hybridization; seroconversion was detected in animals of both species. Conversely, in sheep and horses, virus-specific antibodies did not develop and no evidence of viral replication in the upper respiratory tract was found. These results prove the susceptibility of llamas and pigs to MERS-CoV infection. Thus, the possibility of MERS-CoV circulation in animals other than dromedaries, such as llamas and pigs, is not negligible.

Middle East respiratory syndrome (MERS) coronavirus (MERS-CoV) first emerged in 2012 in Saudi Arabia and is currently a worldwide concern (*1*). As of September 21, 2016, the World Health Organization had confirmed ≈1,800 MERS cases and 643 associated deaths (*2*). Although during most reported outbreaks the virus is mainly transmitted by human-to-human contact, infection through contact with dromedary camels (*Camelus dromedaries*) plays a major role in the primary cases. In the Middle East and some countries from East Africa where MERS is endemic, high prevalence of MERS-CoV–specific antibodies in dromedaries has been reported (*3**–**7*). Moreover, a recent surveillance study in Saudi Arabia demonstrated that MERS-CoV strains isolated from humans were also detected in the upper respiratory tract of dromedaries of several geographic origins, indicating that the virus did not require mutations to jump between species (*8*). However, not all index cases can be explained by direct contact with dromedaries, and transmission from other domestic livestock or animal species to humans cannot yet be ruled out. Recently, evidence that alpacas (*Vicugna pacos*) were also susceptible to MERS-CoV infection was provided and confirmed by field studies performed in Qatar (*9**–**11*). In contrast, despite the ability of the virus to infect a plethora of cell lines and tissues from mammals of multiple species in vitro (*12*), serologic surveys of ruminants and horses did not conclusively determine circulation of MERS-CoV among these domestic animals (*6**,**13**,**14*). Sampling design could explain negative results, and experimental infections provide, in many instances, a more straightforward answer to virus host susceptibility. This knowledge is crucial for determining risk factors with regard to possible globalization of the disease.

Our aim with this study was to address these critical research gaps and to understand the potential role that other animals (besides dromedaries and alpacas) could play in MERS-CoV dissemination. We experimentally inoculated MERS-CoV into llamas (*Lama glama*), pigs (*Sus scrofa*), horses (*Equus ferus caballus*), and sheep (*Ovis aries*). We based our selection of species on epidemiologic interest and on sequence similarities in the MERS-CoV receptor binding domain of dipeptidyl peptidase-4 (DPP4).

## Materials and Methods

### Ethics

All experiments with MERS-CoV were performed at Biosafety Level 3 (BSL-3) facilities of the Biocontainment Unit of the Institut de Recerca i Tecnologia Agroalimentàries (IRTA) Centre de Recerca en Sanitat Animal (CReSA) in Barcelona, Spain. The study was approved by the Ethical and Animal Welfare Committee of IRTA and the Ethical Commission of Animal Experimentation of the Autonomous Government of Catalonia.

### Cells and MERS-CoV

Vero cells were cultured in Dulbecco modified Eagle medium (DMEM; Lonza, Basel, Switzerland) supplemented with 1% fetal calf serum (EuroClone, Pero, Italy), 100 U/mL penicillin, 100 mg/mL streptomycin, and 2 mmol/L glutamine. Passage 7 human isolate MERS-CoV stock (HCoV-EMC/2012) was propagated in Vero cells at 37°C in a CO_2_ incubator for 3 days. The infectious virus titer was determined in Vero cells and calculated by determining the dilution that caused cytopathic effect in 50% of the inoculated cell cultures (50% tissue culture infectious dose endpoint [TCID_50_]).

### Animal Studies

All animals used in this study were obtained from France and Spain by private sale and housed at BSL-3 animal facilities (IRTA-CReSA, Barcelona, Spain). We obtained 8 llamas (6–8 months of age), 8 horses (6–8 months), 14 sheep (2–3 months), and 14 pigs (2 months). We intranasally inoculated a 10^7^ TCID_50_ dose in 3 mL saline solution into each animal (1.5 mL in each nostril) by using a mucosal atomization device (LMA; North-America, Inc., San Diego, CA, USA) and monitored the animals daily for clinical signs (sneezing, coughing, nasal discharge, dyspnea). Rectal temperatures were recorded with a fast display digital thermometer (Digi-Temp; Kruuse Veterinary Products, Langeskov, Denmark) until postinoculation day 10. Nasal and rectal swabs were obtained on postinoculation days 1, 2, 3, 4, 7, 10, 14, and 24 in phosphate-buffered saline (PBS) (for PCR analysis) and DMEM (for virus isolation and titration) containing antimicrobial drugs (100 U/mL penicillin and 0.1 mg/mL streptomycin). All samples were stored at −80°C until tested. Serum samples were obtained before challenge and at postinoculation days 14 and 24 and were subsequently used to detect MERS-CoV–specific antibodies.

### Virus Titration

Nasal swabs collected at different times after inoculation were evaluated for infectious virus by titration in Vero cells. We prepared 10-fold dilutions, starting with a dilution of 1:10, and transferred the dilutions to Vero cells. Plates were monitored daily under a light microscope, and wells were evaluated for cytopathic effect. The final determination was conducted on postinoculation day 5. The amount of infectious virus in swab samples was calculated by determining the TCID_50_.

### Pathology Studies

On postinoculation day 2, we euthanized 4 pigs and 4 sheep with an overdose of pentobarbital followed by exsanguination; using the same procedure, on postinoculation day 4, we euthanized 4 animals of each species (including llamas and horses) and on postinoculation day 24, the remaining animals. We performed complete necropsies and collected respiratory tissues (frontal, medial, and caudal turbinates; proximal, medial and distal trachea; large and small bronchi; and right cranial, mediodorsal, and caudal lung parenchyma) for virologic, histopathologic, immunohistochemical (IHC), and in situ hybridization (ISH) examination.

Tissues for pathology studies were fixed by immersion in 10% neutral-buffered formalin, embedded in paraffin, and sectioned at 3 μm for slides. Sequential slides were either stained with hematoxylin and eosin or used to detect the DPP4 receptor and MERS-CoV antigen by IHC and viral genome by ISH (*15**,**16*). In brief, we performed DPP4 IHC staining by using 5 μg/mL of polyclonal goat IgG anti-human DPP4 antibody (R&D Systems, Abingdon, UK) and peroxidase-labeled rabbit anti-goat IgG (1:200; DAKO; Agilent Technologies Company, Santa Clara, CA, USA) as a secondary antibody. To detect MERS-CoV antigen, we used a monoclonal antibody to the nucleocapsid protein (SinoBiological Inc., Beijing, China) as described (*16*). We performed ISH according to the manufacturer’s instructions by using probes targeting the nucleocapsid gene of MERS-CoV (Advanced Cell Diagnostics, Hayward, CA, USA). ISH staining was detected by using the Fast Red substrate as previously reported (*17*). For detection of mucous substances, we stained selected slides (from animals euthanized on postinoculation day 24) with periodic acid–Schiff (PAS) according to standard methods.

### Viral RNA Detection by Reverse Transcription PCR

We collected tissues (0.2–0.5 g) for viral RNA quantification and placed them in cryotubes containing beads and 500 µL DMEM supplemented with 100 U/mL penicillin, 100 mg/mL streptomycin, and 2 mmol/L glutamine. In brief, samples were homogenized at 30 Hz for 2 min by using a TissueLyser II (QIAGEN, Hilden, Germany) and stored at −70°C until use. We extracted viral RNA from nasal swabs, rectal swabs, and tissue homogenates by using a NucleoSpin RNA virus kit (Macherey-Nagel, Düren, Germany) according to the manufacturer’s instructions. The RNA extracts were tested by UpE PCR (*18*). We conducted reverse transcription quantitative PCR (RT-qPCR) by using AgPath-ID One-Step RT-PCR reagents (Applied Biosystems, Foster City, CA, USA) and performed amplification by using a 7500 Fast Real-Time PCR System (Applied Biosystems) programmed as follows: 5 min at 50°C, 20 s at 95°C, and 45 cycles of 3 s at 95°C and 30 s at 60°C. We considered samples with a cycle threshold <40 positive for MERS-CoV RNA.

### MERS-CoV S1 ELISA

We determined specific S1 antibodies in serum samples from postinoculation days 0, 14, and 24 by a MERS-CoV S1 ELISA as previously described (*16*), with some modifications. In brief, 96-well high-binding plates (Sigma-Aldrich, St. Louis, MO, USA) were coated with 100 μL of S1 protein at 1 μg/mL in PBS overnight at 4°C. After blocking with 1% bovine serum albumin/PBS/0.5% Tween20 for 1 h at 37°C, individual serum samples were added at 1:100, followed by 1 h incubation at 37°C. Plates were washed 4 times with PBS, and the species-specific secondary antibody was added. We used the following conjugates: anti-llama IgG:horseradish peroxidase (HRP) (diluted 1:60,000; no. A160–100P, Bethyl Laboratories, Montgomery, TX, USA); anti-pig IgG:HRP (diluted 1:20,000; no. A5670, Sigma-Aldrich); anti-horse IgG(T):HRP (diluted 1: 10,000; no. AAI38P, Bio-Rad, Hercules, CA, USA); and anti-sheep IgG:HRP (diluted 1:10,000; no. 5184–2504, Bio-Rad). After 1 h of incubation at 37°C, wells were washed 4 times with PBS, and TMB (3,3′,5,5′-tetramethylbenzidine) substrate solution was added and allowed to develop for 8–10 min at room temperature, protected from light. We measured optical density at 450 nm.

### MERS-CoV Neutralization Assay

We also tested serum samples collected on postinoculation days 0, 14, and 24 by a specific virus neutralization assay. First, the samples were inactivated at 56°C for 30 min. Following a previous protocol (*16*), we mixed 400 PFU of MERS-CoV (HCoV-EMC/2012) with serial 2-fold dilutions of heat-inactivated serum, incubated the mixture 1 h at 37°C, and inoculated it onto Huh7 cells. The presence of viral antigen was assessed 8 h after inoculation. Cells were fixed with formaldehyde and stained by using rabbit anti–MERS-CoV antibodies and fluorescein isothiocyanate–conjugated swine anti-rabbit immunoglobulins as secondary antibodies. We calculated 90% plaque-reduction neutralization test values for the MERS-CoV neutralization assay.

## Results

### Clinical Signs

After challenge, 3 of 8 llamas showed clinical signs (moderate mucus secretion in 1 nostril) at postinoculation days 4–18 (Technical Appendix Figure 1, panel A). Soon after inoculation, mild excretion of white mucus from the nose was noted for 3 pigs. Minimal mucus excretion in the nose was noted at variable times (postinoculation days 2–16) for 3 horses. We did not detect nasal discharge in any of the sheep during the experiment. No animal of any species showed a significant increase in body temperature after MERS-CoV inoculation (Technical Appendix Figure 1, panel B).

### MERS-CoV RNA and Infectious Virus in Nasal Swab Samples and Viral RNA in Respiratory Tissues

**Figure 1:**
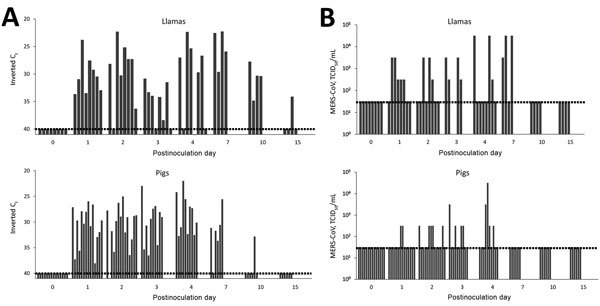
Viral shedding of llamas and pigs after experimental inoculation with MERS-CoV. A) Viral RNA and B) infectious MERS-CoV from nasal swab samples collected from llamas (top) and pigs (bottom) at different times after challenge. Each bar represents an individual animal. Dashed lines depict the detection limit of the assays. C_t_, cycle threshold; MERS-CoV, Middle East respiratory syndrome coronavirus; TCID_50_, 50% tissue culture infective dose.

Pigs and llamas excreted virus in the nose, as evidenced by RT-qPCR of nasal swab samples from postinoculation days 1–10; in 1 llama, viral RNA was detected until postinoculation day 15 (Figure 1, panel A). At postinoculation day 7, the amount of MERS-CoV RNA was still high in all llamas, but a decrease in RNA level was noted at postinoculation day 10. In pigs, high levels of MERS-CoV RNA were demonstrated until postinoculation day 4 and started decreasing at postinoculation day 7; only 1 animal remained positive at postinoculation day 10 (Figure 1, panel A). We subsequently tested positive nasal swab samples for the presence of infectious virus. During the experiment, 7 of 8 llamas and 7 of 14 pigs excreted infectious virus during at least 1 day from postinoculation day 2 on. We detected infectious virus until postinoculation day 4 in pigs and postinoculation day 7 in llamas (Figure 1, panel B). Relatively low levels of viral RNA were detected only until postinoculation day 2 in 5 of 8 inoculated horses and only at postinoculation day 1 in 7 of 14 sheep, suggesting the presence of residual inoculum in these animals (data not shown). We did not detect virus in rectal swab samples from any animal.

**Figure 2:**
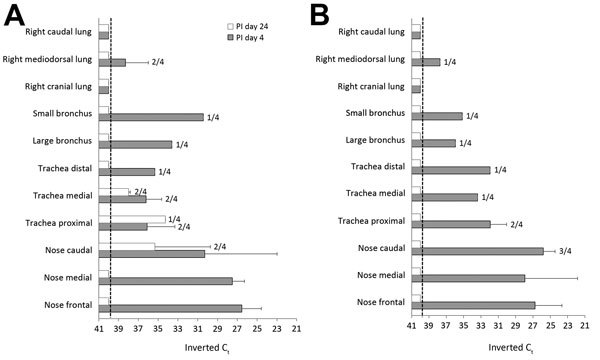
MERS-CoV viral RNA in respiratory tissues of llamas (A) and pigs (B). Viral RNA was determined in tissue homogenates at postinoculation days 4 and 24. Error bars indicate SDs when results were positive in >1 animal. Dashed lines depict the detection limit of the assays (C_t_ ≤40). C_t_, cycle threshold; MERS-CoV, Middle East respiratory syndrome coronavirus; PI, postinoculation.

To determine the presence of viral RNA in tissues, we euthanized representative numbers of animals on postinoculation days 2 (pigs), 4 (llamas and pigs), and 24 (llamas and pigs) and tested tissue homogenates by RT-qPCR. Early after infection, virus RNA transcripts were detected mainly in the nose, trachea, and small and large bronchi of llamas and pigs (Figure 2). At postinoculation day 24, viral RNA was detected only in some tissues (caudal nose and trachea) in 2 llamas (Figure 2).

### Pathology, IHC, and ISH

We observed no substantial macroscopic changes attributable to MERS-CoV infection in any animals. All horses exhibited purulent inflammation of the guttural pouch (empyema), which was most likely of bacterial origin.

**Figure 3:**
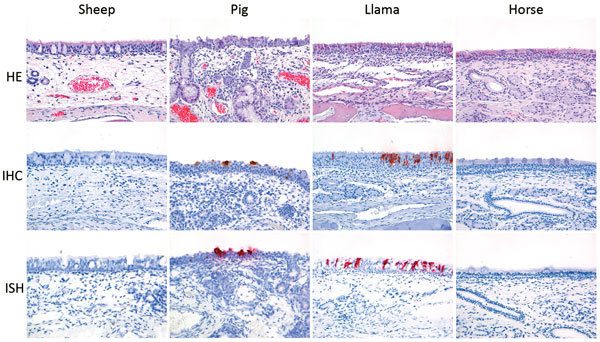
Histology and expression of viral antigen (IHC) and viral RNA (ISH) at postinoculation day 4 in the nasal respiratory epithelium of sheep, pigs, llamas, and horses inoculated with MERS-CoV. A mild to severe rhinitis with epithelial necrosis and hypertrophy and inflammation of the epithelium and lamina propria was observed in the nasal respiratory tissue of pigs and llamas. Associated with these was presence of virus antigen (IHC) and RNA (ISH). No substantial lesions, virus antigen, or virus RNA were detected in the nasal respiratory tissues of sheep and horses (HE, IHC, ISH). Original magnification ×200 for all images. HE, hematoxylin and eosin; IHC, immunohistochemistry; ISH, in situ hybridization; MERS-CoV, Middle East respiratory syndrome coronavirus.

At postinoculation day 4, IHC and ISH demonstrated virus antigen and nucleic acid in a few nasal respiratory epithelial cells in 3 of 4 llamas and in 2 of 4 pigs (Figure 3). At postinoculation day 2, occasional alveolar epithelial cells were positive for antigen and nucleic acid in 1 of 4 pigs. Also at postinoculation day 2, occasional bronchiolar cells from 1 of 4 sheep were positive for MERS-CoV (Technical Appendix Figure 2). MERS-CoV antigen was absent in the rest of the respiratory tissues of animals of all species collected on any of the days. Associated with the presence of virus antigen and nucleic acid, llamas and pigs demonstrated a mild to moderate rhinitis characterized by mild to moderate epithelial necrosis with infiltration of variable numbers of neutrophils in the epithelium (exocytosis) and in the lumen and mild to severe infiltration of the lamina propria with variable numbers of macrophages, lymphocytes, neutrophils, and plasma cells and multifocal mild edema. We also observed mild to moderate multifocal epithelial hypertrophy consistent with regeneration (Figure 2). We observed no other relevant microscopic lesions in horses (besides the evidence of purulent empyema) and sheep.

### DPP4 Receptor Distribution and Presence of Mucosubstances in Respiratory Tissues 

**Figure 4:**
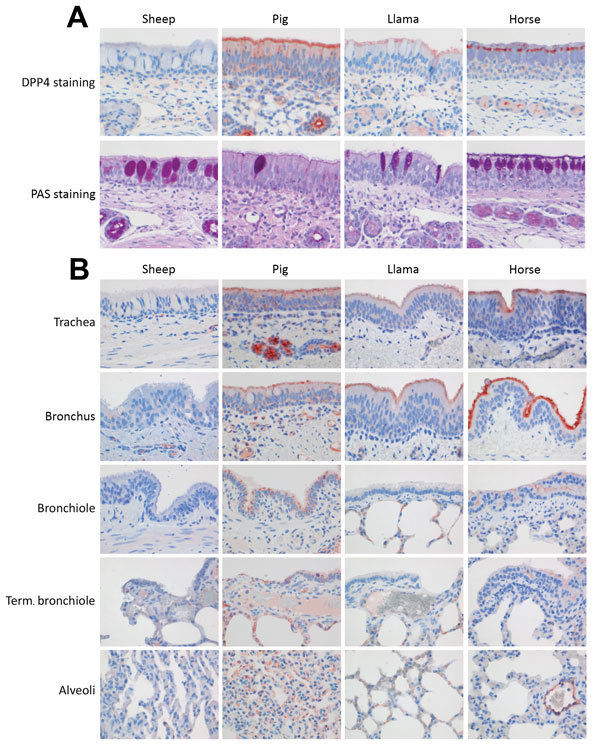
Presence of MERS-CoV receptor DPP4 (IHC) and of mucosubstances (PAS) in upper and lower respiratory tract tissues from sheep, pigs, llamas, and horses. A) In the nose, DPP4 (red cytoplasmic or membrane staining) was present on the lining epithelium of pigs, llamas, and horses but not sheep. PAS staining (magenta) demonstrated more mucous cells in the lining epithelium of sheep and horses and a layer of mucus on the lining epithelium of the horses. B) DPP4 (red cytoplasmic or membrane staining) was present on the lining epithelium of the trachea, bronchus/bronchioles, and alveoli in the pigs, llamas and horses but not in the sheep. Original magnification ×400 for all images. DPP4, dipeptidyl peptidase-4; IHC, immunohistochemistry; MERS-CoV, Middle East respiratory syndrome coronavirus; PAS, periodic acid–Schiff; term., terminal.

DPP4 IHC staining of upper and lower respiratory tract tissues collected on postinoculation day 24 found DPP4 expression on the respiratory epithelium of the nose of llamas, pigs, and horses but only to a very limited extent on that of sheep (Figure 4, panel A). We also detected DPP4 expression in the lower respiratory tract (but restricted to tracheal and bronchial epithelia) of horses, llamas, and pigs (Figure 4, panel B). PAS staining demonstrated the presence of mucosubstances in the nose of llamas, pigs, horses, and sheep, with a relatively higher number of mucous (goblet) cells in the lining epithelium of sheep and horses. In the horses only, there was also a layer of mucus covering lining epithelium with a multifocal distribution.

### Specific Humoral Immune Response

**Figure 5:**
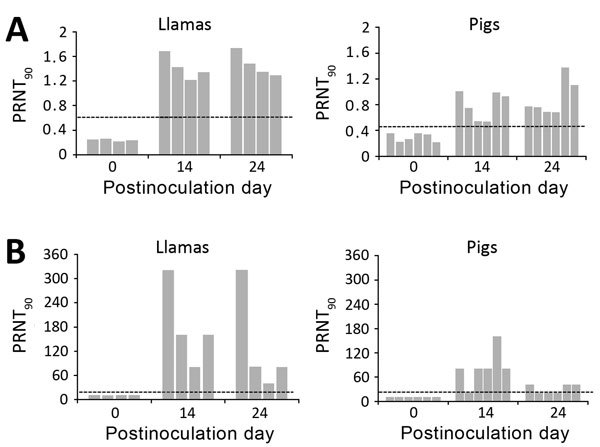
Antibody responses after experimental inoculation of MERS-CoV into llamas and pigs. A) MERS-CoV S1 antibody responses were analyzed in serum from all animals at postinoculation days 0, 14, and 24. An ELISA with recombinant MERS-CoV S1 protein was used, and results are represented individually. B) Individual MERS-CoV neutralization titers from llamas and pigs as determined from serum. Dashed lines depict the detection limit of the assays. MERS-CoV, Middle East respiratory syndrome coronavirus; OD, optical density; PRNT_90_, 90% plaque reduction neutralization test.

ELISA results showed antibodies against the S1 protein in all llamas and pigs from postinoculation day 14 on, although the response in pigs was weaker than that in llamas (Figure 5, panel A). We confirmed the specificity of the response by virus neutralization assay (Figure 5, panel B). In all llamas, serum neutralizing MERS-CoV-specific antibody titers (1:80 to 1:320) were detected at postinoculation days 14 and 24. In addition, 14 days after challenge, in 5 of 6 pigs, MERS-CoV neutralizing antibodies were detected (1:80 to 1:160). However, 10 days later, these virus neutralizing antibodies decreased (1:20 to 1:40) (Figure 5, panel B). We detected no MERS-CoV–specific antibodies in serum of sheep and horses.

## Discussion

Our study results indicate that pigs and llamas are susceptible to MERS-CoV infection. These animals shed infectious virus until postinoculation days 4 (pigs) and 7 (llamas), although titers were lower among pigs. In pigs and llamas, as well as dromedary camels (*16*) and alpacas (*9*), we detected virus predominantly in the nasal respiratory epithelium by IHC, ISH, or both. Accordingly, we mainly detected viral RNA, as assessed by RT-qPCR, in the nose, trachea, and bronchi of those animals. We also detected viral RNA in lung tissue from 2 of 4 pigs euthanized at postinoculation day 2. Virus shedding in dromedary camels and alpacas for longer periods, up to 14 days after experimental inoculation, has been reported (*9**,**16**,**19*). Of note, the level of MERS-CoV excreted in the nose of dromedaries seems to be much higher (*16**,**19*) than that of other animal species described so far, suggesting a more prominent role of dromedaries in transmission of MERS-CoV to humans.

Differences in virus susceptibility and pathogenicity between animals of different species could be explained by a distinct tissue distribution of DPP4, the MERS-CoV receptor. In our study, DPP4 distribution in the respiratory tract was similar among llamas and pigs but differed from that of dromedary camels (*15*). In contrast, DPP4 was barely detected in the respiratory tract of sheep, probably accounting for the lack of infection reported here. These results are in concordance with those reported by Adney et al., that MERS-CoV experimentally inoculated sheep showed no clinical disease and that only small amounts of virus were detected in nasal swab samples (*20*). Differences in susceptibility to MERS-CoV infection and level of virus excretion might also result from host factors associated with innate immunity. Surprisingly, horses were not susceptible to MERS-CoV despite high expression and wide distribution of the virus receptor along the respiratory tract. Moreover, the receptor binding domain, and in particular key amino acids on the docking site, are identical in horses and humans (*21*). Although human, camel, and horse DPP4 served as potent and nearly equally effective MERS virus receptors (*22*), horses were not productively infected by the strain of MERS-CoV used in this study. Detection of low levels of viral RNA in nasal swab samples until postinoculation day 2 can be attributed to residual inoculum. Similarly, a recent study with horses also showed low levels of MERS-CoV excretion and no virus neutralizing antibodies (*20*). 

These results highlight that other mechanisms, such as epithelial cell permissibility or strong innate immune responses, may influence the establishment of infection. In that respect, PAS staining revealed differences in the number of goblet cells in the lining epithelium and mucus covering epithelial surfaces, which may have impeded the binding of the virus to the respiratory epithelium of horses. Also, virus tends to bind more to ciliated or nonciliated non–mucus-producing cells and, in proportion, these cells may be fewer in horses than in llamas and pigs. However, it is possible that the guttural pouch empyema, which most likely was of bacterial origin (probably *Streptococcus* spp.), may have influenced mucus production in the horses. Although these observations are in line with those from studies in the field indicating the absence of antibodies to MERS-CoV in equids (*14*), this aspect should be studied further.

Epidemiologic studies have provided evidence of endemic MERS-CoV infection among dromedaries in the Greater Horn of Africa as far back as 1983 (*23**,**24*) and in Saudi Arabia as far back as 1992–1993 (*25*). To implement optimal serologic surveillance in countries where MERS is and is not endemic, identifying which animal species might be potential reservoirs for MERS-CoV, besides dromedaries, is crucial. The finding that pigs can be infected with MERS-CoV suggests that other members of the family Suidae could be susceptible to the virus, such as common warthogs (*Phacochoerus africanus*), bushpigs (*Potamochoerus larvatus*), and wild boars (*Sus scrofa scrofa*). Indeed, these animals are commonly found in the Greater Horn of Africa or the Middle East, sharing territories and water sources with dromedaries. Thus, members of the family Suidae might merit inclusion in MERS surveillance programs. Further studies need to be done to investigate MERS-CoV transmission within and among species to provide a better understanding of the role of potential reservoirs during an outbreak. Moreover, studies comparing the innate immunity of horses with susceptibility of other animal species (i.e., dromedary camels, alpacas, llamas, or pigs) are needed.

Technical Appendix Clinical signs in llamas, pigs, horses, and sheep and histologic, immunohistochemical, and in situ hybridization findings in sheep after inoculation of Middle East respiratory syndrome coronavirus.
