# Supplementary material for: Livestock Susceptibility to Infection with Middle East Respiratory Syndrome Coronavirus
Source: Emerg Infect Dis. 2017 Feb;23(2):232–40. doi: 10.3201/eid2302.161239 (PMC5324816; doi:10.3201/eid2302.161239)
Supplement: Technical Appendix — Clinical signs in llamas, pigs, horses, and sheep and histologic, immunohistochemical, and in situ hybridization findings in sheep after inoculation of Middle East respiratory syndrome coronavirus. [file 16-1239-Techapp-s1.pdf]

Article DOI: http://dx.doi.org/10.3201/eid2302.161239

Livestock Susceptibility to Infection with Middle East Respiratory Syndrome Coronavirus

Technical Appendix

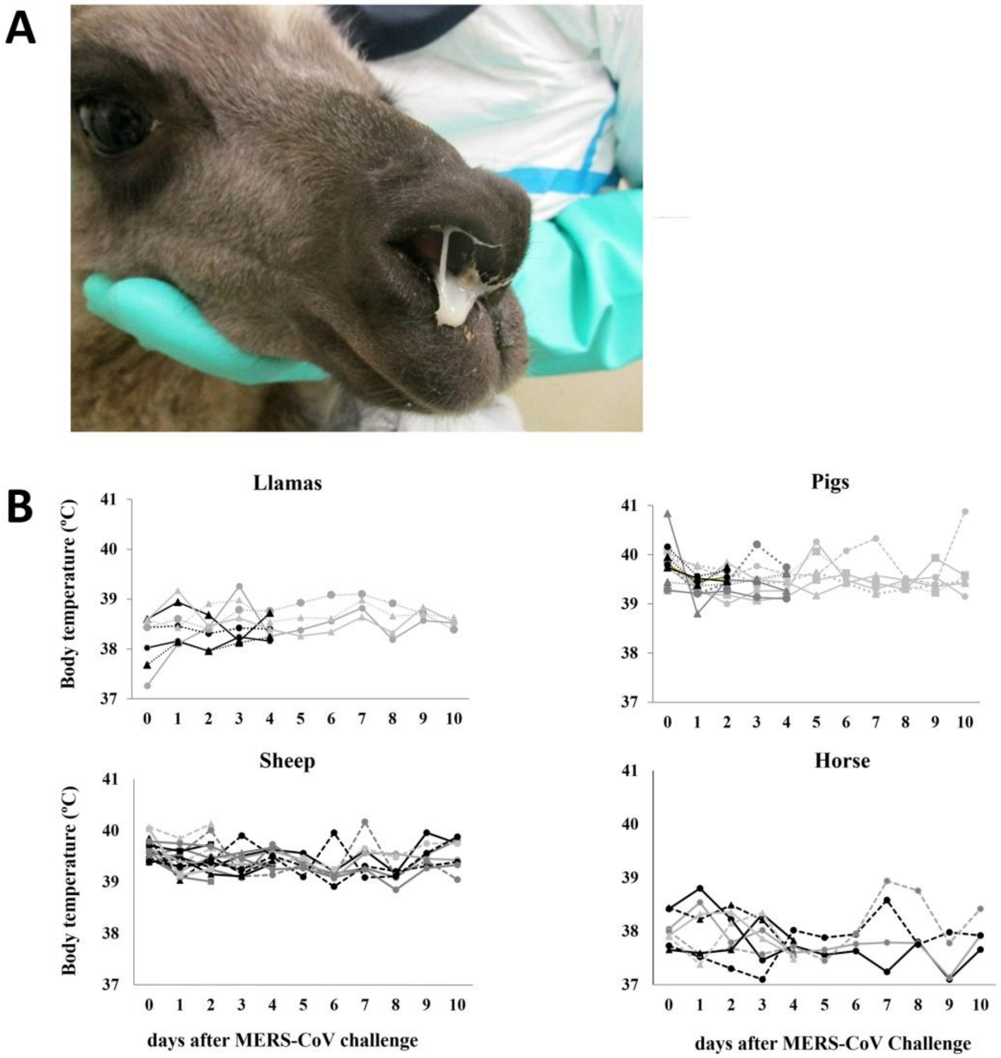

Technical Appendix Figure 1. Clinical signs after MERS-CoV inoculation in llamas, pigs, horses, and sheep. A) Presence of mucus excretion in a llama at 4 days postinoculation. B) Rectal body temperatures in llamas, pigs, sheep, and horses at different times postinoculation.

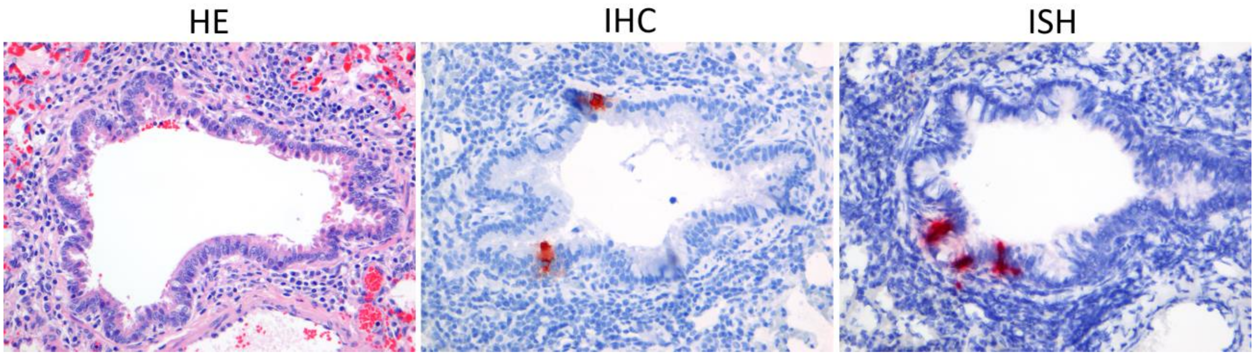

Technical Appendix Figure 2. Histology, IHC, and ISH in the lung of a sheep 2 days postinoculation with MERS-CoV. Few bronchiolar cells stained positive for MERS-CoV antigen in 1 out of 4 sheep (HE, IHC, ISH magnification ×200).
